# Production of l-lactic acid by the yeast *Candida sonorensis* expressing heterologous bacterial and fungal lactate dehydrogenases

**DOI:** 10.1186/1475-2859-12-53

**Published:** 2013-05-25

**Authors:** Marja Ilmén, Kari Koivuranta, Laura Ruohonen, Vineet Rajgarhia, Pirkko Suominen, Merja Penttilä

**Affiliations:** 1 VTT Technical Research Centre of Finland, Espoo, Finland; 2 Cargill Biotechnology Research and Development, Minnesota, USA; 3 Present address: Total Gas and Power Biotech USA, Emeryville, California, USA

## Abstract

**Background:**

Polylactic acid is a renewable raw material that is increasingly used in the manufacture of bioplastics, which offers a more sustainable alternative to materials derived from fossil resources. Both lactic acid bacteria and genetically engineered yeast have been implemented in commercial scale in biotechnological production of lactic acid. In the present work, genes encoding l-lactate dehydrogenase (*LDH*) of *Lactobacillus helveticus*, *Bacillus megaterium* and *Rhizopus oryzae* were expressed in a new host organism, the non-conventional yeast *Candida sonorensis,* with or without the competing ethanol fermentation pathway.

**Results:**

Each *LDH* strain produced substantial amounts of lactate, but the properties of the heterologous *LDH* affected the distribution of carbon between lactate and by-products significantly, which was reflected in extra-and intracellular metabolite concentrations. Under neutralizing conditions *C. sonorensis* expressing *L. helveticus LDH* accumulated lactate up to 92 g/l at a yield of 0.94 g/g glucose, free of ethanol, in minimal medium containing 5 g/l dry cell weight. In rich medium with a final pH of 3.8, 49 g/l lactate was produced. The fermentation pathway was modified in some of the strains studied by deleting either one or both of the pyruvate decarboxylase encoding genes, *PDC1* and *PDC2*. The deletion of both *PDC* genes together abolished ethanol production and did not result in significantly reduced growth characteristic to *Saccharomyces cerevisiae* deleted of *PDC1* and *PDC5*.

**Conclusions:**

We developed an organism without previous record of genetic engineering to produce L-lactic acid to a high concentration, introducing a novel host for the production of an industrially important metabolite, and opening the way for exploiting *C. sonorensis* in additional biotechnological applications. Comparison of metabolite production, growth, and enzyme activities in a representative set of transformed strains expressing different *LDH* genes in the presence and absence of a functional ethanol pathway, at neutral and low pH, generated a comprehensive picture of lactic acid production in this yeast. The findings are applicable in generation other lactic acid producing yeast, thus providing a significant contribution to the field of biotechnical production of lactic acid.

## Background

A variety of new products based on polymerized lactic acid are constantly being developed, increasing the demand for lactic acid. L-Lactic acid is typically produced in large quantities by carbohydrate fermentation by lactic acid bacteria. The fermentation is efficient at near neutral pH, controlled with neutralizing chemicals and generating lactate salts [1]. The undissociated (free) lactic acid rather than the salt of the acid is the required product for the polymerization reaction and additional processing is necessary to recover free lactic acid. Yeast are considered as attractive alternative hosts for lactic acid production at low pH because they are more acid tolerant than lactic acid bacteria. Low pH production would decrease the need for neutralizing chemicals. Several groups have demonstrated efficient production of l*-*lactic acid by *S. cerevisiae* expressing a heterologous gene encoding lactate dehydrogenase (*LDH*) [2-4]. The *LDH* gene has also been introduced into some non-conventional yeast species that have advantageous characteristics such as good acid tolerance or ability to metabolize carbohydrates that *S. cerevisiae* does not naturally consume. For example, *Kluyveromyces lactis*[5,6], *Pichia stipitis*[7], *Candida boidinii*[8] and *Candida utilis*[9] have been shown to produce high concentrations of lactic acid. In addition, e.g. *Zygosaccharomyces bailii*[10]*,* and *Kluyveromyces marxianus*[11] expressing *LDH* have been shown to produce lactic acid.

One of the main issues related to lactic acid production using yeast, especially *S. cerevisiae*, is the ability of the yeast to produce ethanol in the presence of excess glucose. Even though the expression of the lactate dehydrogenase gene can itself decrease the conversion of glucose to ethanol to some extent [12], modification of the ethanol pathway, to remove competition with lactate dehydrogenase for pyruvate, has proved an effective way to increase the yield of lactic acid on glucose [2]. A single deletion of the pyruvate decarboxylase gene *PDC1*, encoding the main PDC isoenzyme in *S. cerevisiae*, decreased PDC activity moderately but the expression of *PDC5* was enhanced in the absence of PDC1 [13,14]. A double deletion of *PDC1* and *PDC5* in lactic acid producing *S. cerevisiae* strains decreased ethanol production and increased lactic acid yield significantly, but still some ethanol was produced because the *PDC6* gene was intact [2]. In addition, the growth of the *PDC1* and *PDC5* deleted strain was severely reduced on glucose medium [2], which may be undesirable in a production process. In contrast, the deletion of the only pyruvate decarboxylase encoding gene, *PDC1*, from *K. lactis* had only a mild effect on growth, was sufficient to eliminate ethanol production and improve lactate production [6].

Efficiency of lactic acid production will be affected not only by the choice of the host strain but also to some extent by the enzymatic properties of different LDH enzymes. *LDH* genes from different organisms result in different LDH activity levels and concentrations of produced lactic acid when expressed in the same *S. cerevisiae* host strain [4,10,15]. LDH activity level was also affected by the copy number of the *LDH* gene in the host [16].

We developed vectors and techniques for introducing genetic modifications into the non-conventional yeast *C. sonorensis* which enabled its genetic engineering for the first time. *C. sonorensis* is a methylotrophic yeast that readily ferments glucose to ethanol, utilizes several carbon sources including the pentose sugars xylose and arabinose, is relatively tolerant to acidic conditions, and has simple nutritional requirements [17,18]. The objective of the present work was to construct *C. sonorensis* strains expressing a heterologous *LDH* gene and containing an intact or modified ethanol fermentation pathway, and to characterize the effects of these modifications on lactic acid production. Strains expressing the l-lactate dehydrogenase encoding genes from *Lactobacillus helveticus*, *Bacillus megaterium*, and from the fungus *Rhizopus oryzae* were compared and evaluated for their relative efficiency in lactate production by *C. sonorensis.* The effect of increased LDH activity level as a result of expressing multiple *LDH* gene copies per genome was determined in strains containing a functional ethanol pathway and in strains deleted of the *PDC* genes. These studies revealed that production of lactate, ethanol and pyruvate was determined by the *PDC* modifications, the choice of LDH enzyme, and the LDH enzyme activity level, which varied with the *LDH* gene copy number.

## Results

### Development of tools for *C. sonorensis* transformation

Growth inhibition tests in YPD medium supplemented with antibiotics in a range of concentrations suggested that ≥200 μg/ml of G418 was inhibitory and thus could probably be used for the selection of transformants. Furthermore, *C. sonorensis* was melibiase (α-galactosidase) negative suggesting that transformants could be selected based on growth on the disaccharide melibiose, or screened on the chromogenic substrate X-α-gal.

Initial attempts to transform *C. sonorensis* with pTEF/Zeo, pMI203 and pMI205, containing the zeocin resistance gene expressed under heterologous promoters, did not yield selectable transformants. For this reason, a genomic library was constructed to isolate *C. sonorensis* promoters to direct the expression of *LDH* and marker genes. Genes encoding highly expressed glycolytic phosphoglycerate kinase (*PGK1*) and glyceraldehyde-3-phosphate dehydrogenase (*TDH1*) were isolated by hybridization with the *C. albicans PGK1* and the *S. cerevisiae TDH1* probes, respectively. Sequences upstream of the predicted open reading frames (*i.e*. promoters) of the *PGK1* and *TDH1* genes were subsequently cloned upstream of the ORFs of the marker genes *MEL5* and G418^R^. *C. sonorensis* was successfully transformed with each of the four linearized constructs using the lithium acetate method. Both the direct selection for *MEL5*-containing transformants on minimal medium containing melibiose as the sole carbon source and the detection of blue colour on non-selective X-α-gal plates were suitable methods for the isolation of transformants. Southern analyses indicated that the integration sites varied between the transformants (data not shown).

### Isolation of *PDC1* and *PDC2* and demonstration of their functional roles in ethanol production

A 0.6 kb fragment of a *PDC* sequence homologue was amplified by PCR from *C. sonorensis* DNA using degenerate primers for *PDC.* The fragment was used as a probe to isolate the corresponding full length *PDC1* gene (acc. AM420319) from the genomic library. Additional PCR reactions with the same degenerate *PDC* primers revealed another putative *PDC* sequence present in *C. sonorensis*, and a full length *PDC2* gene (acc. AM420320) was isolated. The predicted open reading frames of the *PDC1* and *PDC2* genes code for 575 and 568 amino acids, respectively, and have 62% amino acid sequence identity to each other, 68% and 59% identities with *Ogataea parapolymorpha* PDC (acc. EFW96140.1), and 61% and 63% identities with *Candida boidinii* PDC1 (acc. BAI43440), respectively, as the best hits identified in database searches (BLASTP 2.2.26). This supports the hypothesis that the two genes code for pyruvate decarboxylases.

**Figure 1:**
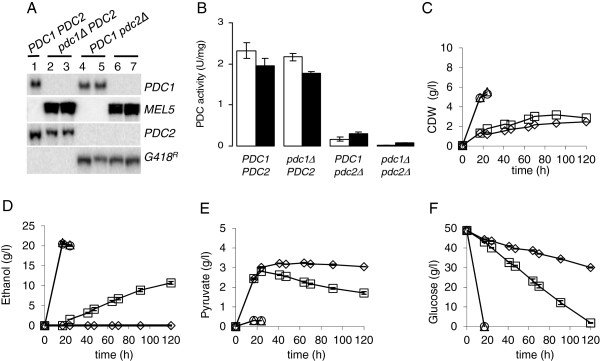
**Characterization of *PDC *knock-out strains.** (**A**) Southern analysis of *C. sonorensis* wild type, *pdc1*∆, *pdc2*∆, and *pdc1*∆ *pdc2*∆ strains with *PDC1, PDC2, MEL5, and G418*^*R*^ probes. (**B**) PDC enzyme activity in *C. sonorensis* transformants expressed as units per mg protein (U/mg). Lower case letters indicate gene deletion and upper case letters the presence of an intact *PDC* gene. Activity was measured at 20 h (white columns) and 40 h (black columns) from cultures grown on YP +% (w/v) glucose medium. Data are means ± SEM (n=2). Biomass (**C**) and concentrations of ethanol (**D**), pyruvate (**E**), and glucose (**F**) of *C. sonorensis* wild type (○), *pdc1*∆ (∆), *pdc2*∆ (□), and *pdc1*∆ *pdc2*∆ (⋄) strains in cultures grown on YP + 5% (w/v) glucose medium. Data are means ± SEM (n=3-4). Where no error bars are seen, SEM was less than the size of the symbol.

To assess the functional role of the two *PDC* genes, strains deleted of either one or both *PDC* genes were constructed. *C. sonorensis PDC1* was replaced by the *MEL5* marker using pMI267, while *PDC2* was replaced by the *G418*^R^ marker using pMI287. Screening of transformants for decreased ethanol production enabled detection of candidate *PDC2*-deleted strains, but candidate *PDC1*-deleted strains could not be distinguished. Southern analyses were used to screen for *PDC1*-deletion and to confirm *PDC2* deletion by the absence of *PDC1*- or *PDC2*-specific hybridization signals (Figure 1A) and the appearance of transformation marker–specific signals of appropriate size. Transformants deleted of *PDC1* or *PDC2* were found at 15% and 5% frequency, respectively.

The parent and the various *PDC* deletion strains were grown in YP-5% glucose and pyruvate decarboxylase enzyme activity was measured. PDC activity was similar in the wild type and *pdc1*∆ strains, but was reduced to 20% of the wild type activity or lower in the *pdc2*∆ strain. The *pdc1*∆ *pdc2*∆ strain had essentially no detectable PDC activity (Figure 1B).

Deletion of *PDC1* or *PDC2* affected ethanol production differently. Strains with an intact *PDC2* produced similar amounts of ethanol irrespective of the presence or absence of *PDC1* (Figure 1D). Deletion of *PDC2* alone caused a large decrease in ethanol production, but deletion of both *PDC1* and *PDC2* was necessary to eliminate ethanol production (Figure 1D). These data, together with the enzyme activity measurements (Figure 1B) and Northern analyses on the expression of *C. sonorensis PDC1* and *PDC2* genes (data not shown) demonstrate that *PDC2* codes for a PDC isoenzyme that is abundant and is the main enzyme responsible for directing pyruvate to acetaldehyde and further to ethanol production.

The *pdc1*∆ *pdc2*∆ and *pdc2*∆ strains excreted significantly more pyruvate than strains with an intact *PDC2* (Figure 1E). However, the *pdc2*∆ strain consumed pyruvate when ethanol was being produced, while no net reduction in pyruvate concentration was observed with the ethanol non-producing *pdc1*∆ *pdc2*∆ strain.

The *pdc1*∆ *pdc2*∆ strains utilized glucose the slowest (Figure 1F), the two strains with an intact *PDC2* the fastest, and *pdc2*∆ showed an intermediate glucose consumption rate. *PDC2* deletion also resulted in an approximately 50% decrease in the final biomass, while *PDC1* deletion did not affect biomass accumulation (Figure 1C).

### Lactate and ethanol production with strains expressing different *LDH* genes

*LDH* genes from three different sources, *L. helveticus, B. megaterium* and *R. oryzae,* were separately expressed in *C. sonorensis* under control of the *C. sonorensis PGK1* promoter. Integration of the *LDH* gene was targeted into the *PDC1* locus to provide a uniform set of strains, which produce both ethanol and lactic acid, for comparison.

**Figure 2:**
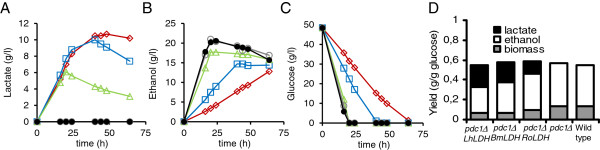
**Comparison between *pdc1Δ::LhLDH*, *pdc1Δ::BmLDH*, and *pdc1Δ::RoLDH *strains.** Lactate (**A**), ethanol (**B**) and glucose (**C**) concentrations in YP+ 5% (w/v) glucose medium by *pdc1Δ::LhLDH* (⋄), *pdc1Δ::BmLDH* (□), *pdc1Δ::RoLDH* (∆) strains, the *pdc1Δ* strain without *LDH* (○), and wild type *C. sonorensis* (●). The final yields of lactate, ethanol and biomass on glucose, determined at the sample time when glucose was exhausted, are shown in panel D. Data are means ± SEM (n= 3–9). Where no error bars are seen, SEM was less than the size of the symbol.

Strains expressing *LhLDH* (pMI257 transformants), *BmLDH* (pMI265 transformants), *RoLDH* (pMI266 transformants) or no *LDH* (pMI267 transformants), produced different amounts of lactate, ethanol and biomass and consumed glucose at different rates (Figure 2). The *pdc1Δ::LhLDH* and *pdc1Δ::BmLDH* strains produced similar lactate concentrations, whereas the *pdc1Δ::RoLDH* strain produced significantly less lactate than the two other *LDH* strains (Figure 2A). Ethanol was produced at the highest rate by wild type *C. sonorensis* and the *pdc1Δ* strain without *LDH*, and ethanol production by the *pdc1Δ::RoLDH* strain was only slightly slower (Figure 2B). These strains consumed glucose at a higher rate than the *pdc1Δ::BmLDH* and *pdc1Δ::LhLDH* strains (Figure 2C). The *pdc1Δ::BmLDH* strain produced ethanol and consumed glucose at higher rates than the *pdc1Δ::LhLDH* strain (Figure 2B and C). However, they produced comparable maximum lactate and ethanol concentrations (Figure 2A and B) and final yields on glucose (Figure 2D) even though lactate yield on glucose for the *pdc1Δ::LhLDH* strain was higher than that for the *pdc1Δ::BmLDH* strain during the first 40 h of the cultivation. When lactate production per gram biomass was assessed, the *pdc1Δ::RoLDH* strain was the least and the *pdc1Δ::LhLDH* strain the most efficient in converting glucose to lactate (data not shown). The final biomass produced by the strains lacking *LDH* was higher (OD_600_=22) than that of the *LDH* strains, in particular when compared to the *pdc1Δ::LhLDH* and *pdc1Δ::BmLDH* strains (OD_600_=10) (Figure 2D).

### The effect of multiple *LDH* gene copies on lactate and ethanol production

**Figure 3:**
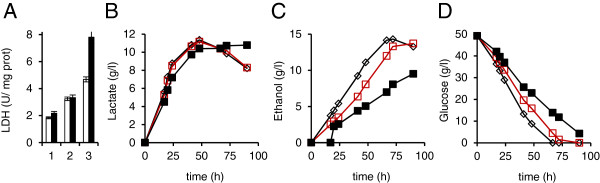
**Effect of additional *LhLDH *copies in *PDC *positive strain background.** (**A**), LDH enzyme activity (U/mg soluble protein) after 20 h (white bars) and 40 h (black bars) cultivation, (**B**), lactic acid (g/l) and (**C**), ethanol (g/l) production, and (**D**), glucose consumption (g/l) in transformants containing 1 (⋄), 2 (□), or 3 (■) copies of the *LhLDH* gene integrated into unknown sites in the *C. sonorensis* genome. The YP+ 5% (w/v) glucose medium was initially inoculated to an OD_600_ of 0.1. Data are means ± SEM (n=2-4). Where no error bars are seen, SEM was less than the size of the symbol.

Strains containing 1 to 3 copies of the *LhLDH* or *BmLDH* gene integrated at non-homologous sites in the genome were identified by Southern analysis (data not shown). LDH enzyme activity increased with increasing *LDH* copy number, but the volumetric lactate production did not increase (data for *LhLDH* shown in Figure 3A and 3B). The yield of lactate on glucose did increase with increasing LDH activity and copy number (e.g. at 48 h 0.28, 0.34, and 0.40 g lactate / g glucose with 1, 2, and 3 LDH copies) owing to significant reduction in ethanol production, glucose consumption (Figure 3C and D) and biomass production (data not shown).

### Comparison of the different *LDH* genes in a *PDC* negative strain background

Representative *PDC1-*deleted strains, each expressing a different *LDH* gene, or no *LDH*, were transformed with the *PDC2* replacement cassette from pMI287 to enable comparison of the different *LDH* strains in the absence of PDC enzyme activity and ethanol production.

**Figure 4:**
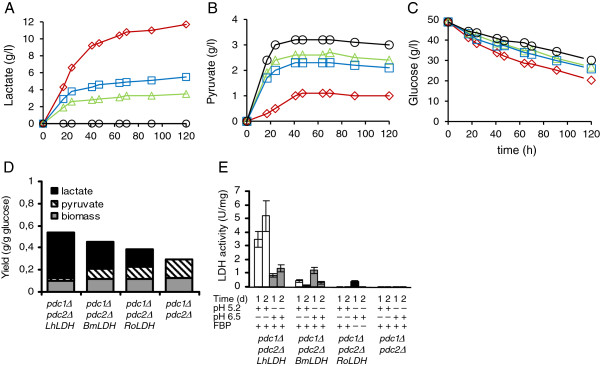
**Comparison between *pdc1Δ::LhLDH pdc2Δ*, *pdc1Δ::BmLDH pdc2Δ*, and *pdc1Δ::RoLDH pdc2Δ *strains.** Lactate (**A**), pyruvate (**B**) and glucose (**C**) concentrations in YP+ 5% glucose medium by the *pdc1Δ::LhLDH pdc2Δ* (⋄), *pdc1Δ::BmLDH pdc2Δ* (□), *pdc1Δ::RoLDH pdc2Δ* (∆) or *pdc1Δpdc2Δ* (○, no *LDH*) strains. (**D**). The final yields of lactate (black), pyruvate (descending diagonal) and biomass (grey) on glucose, determined at 120 h. (**E**). LDH enzyme activities determined at 20 h (1) and 40 h (2). FBP, 5 mM fructose-1, 6-diphosphate. Data are means ± SEM.

The origin of the LDHs had a greater effect on the efficiency of lactate production in the ethanol non-producing *pdc1*∆ *pdc2*∆ transformants (Figure 4) than in the *pdc1*∆ *PDC2* transformants (Figure 3). The *pdc1Δ::LhLDH pdc2Δ* strain produced 2-fold and 3-fold higher lactate concentrations than the *pdc1Δ::BmLDH pdc2Δ* and the *pdc1Δ::RoLDH pdc2Δ* strains, respectively (Figure 4A). Lactate contributed 92% (*LhLDH*), 72% (*BmLDH*) or 59% (*RoLDH*) of the detected extracellular metabolites. All strains excreted pyruvate but the concentrations differed depending on the presence and type of LDH (Figure 4B). The highest pyruvate concentration was produced by the *pdc1*∆ *pdc2*∆ strain without *LDH*. Of the *LDH* strains, the *LhLDH* strains produced the lowest and the *RoLDH* strains the highest pyruvate concentration (Figure 4B and D), analogous to the ethanol concentrations produced by the *pdc1*∆ *PDC2* strains containing the corresponding *LDH* gene (Figure 2B). Introduction of any of the three *LDH* genes enhanced glucose consumption, compared with the *pdc1*∆ *pdc2*∆ strain lacking *LDH*, the *LhLDH* strain being the most efficient in this respect (Figure 4C).

Production of lactate and pyruvate was accompanied with a decrease in the pH of the culture media to pH 3.3 – 3.5 (data not shown).

LDH enzyme activities were measured with or without fructose-1,6-diphosphate at two different pH values due to the differences in the optimal conditions for the individual enzymes [19,20]. The LDH enzyme activity measured *in vitro* in the *PDC* negative strains containing one copy of *LhLDH*, *BmLDH* or *RoLDH* directly correlated with the lactate amount measured; the *LhLDH* strain that produced the highest final lactate concentration also had the highest enzyme activity (Figure 4E).

**Figure 5:**
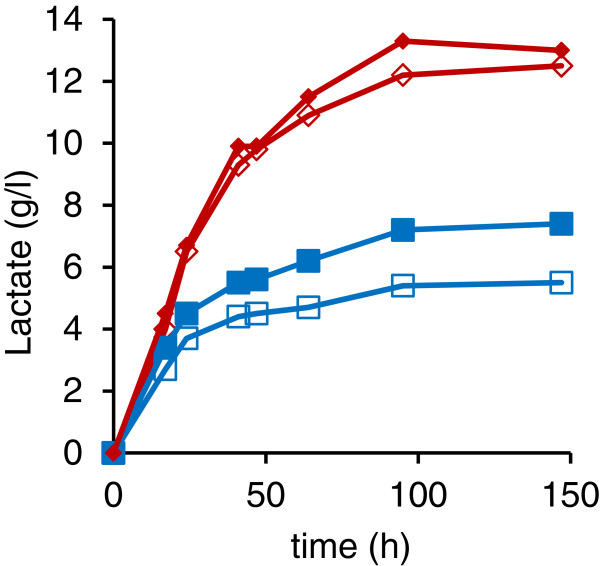
**Effect of a second copy of the *LhLDH *or *BmLDH *gene in *PDC *knock-out strains.** Lactate concentration in YP + 5% glucose medium by the *pdc1Δ::LhLDH pdc2Δ* (⋄), *pdc1Δ::LhLDH pdc2Δ::LhLDH* (♦), *pdc1Δ::BmLDH pdc2Δ* (□), and *pdc1Δ::BmLDH pdc2Δ::BmLDH* (■) strains. Data are means (n=3). SEM was less than the size of the symbol.

Addition of a second *BmLDH* copy, integrated in the *PDC2* locus, increased the final lactate concentration by 30% (Figure 5), reduced pyruvate accumulation by 15%, and enhanced glucose consumption compared to the single copy *BmLDH* strain. Based on these data it appears that the level of BmLDH enzyme activity in the single copy *BmLDH* strain restricted lactate production. In comparison, a second copy of *LhLDH* resulted in small (< 9%) but significant increase in lactate (Figure 5) and decrease in pyruvate production (not shown). Even so, cessation of lactate production still occurred and was not overcome by increasing *LDH* copy number, which suggests that factors other than LDH dosage prevented lactate accumulation in the *C. sonorensis* cultures.

### The correlation to lactate concentration of the concentration of CaCO_3_ added as a neutralizing agent

**Figure 6:**
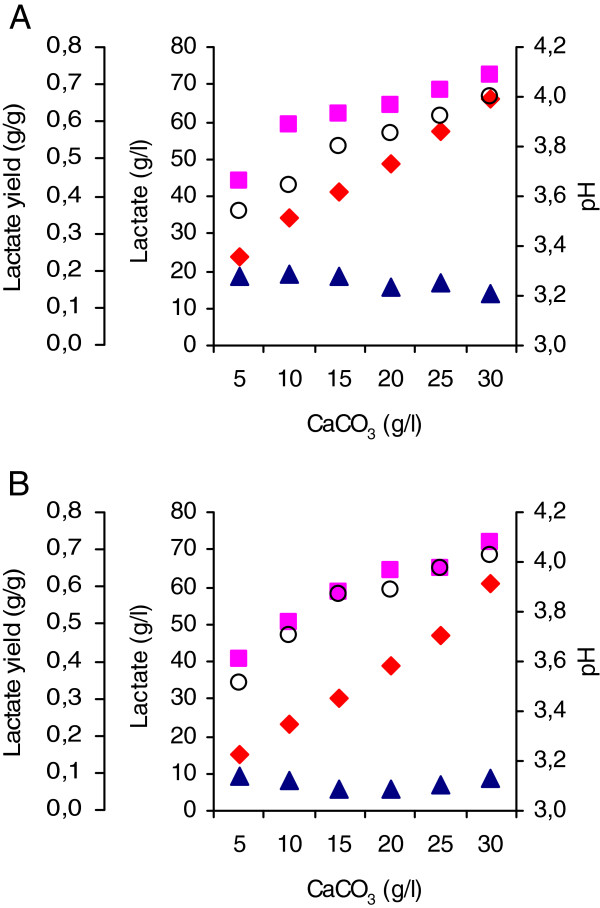
**Effect of CaCO**_**3 **_**concentration of lactate production.** Total concentration of lactate (g/l, ♦), lactate yield (g/g glucose consumed, ■), undissociated lactic acid (g/l, ▲), and pH (○) shown as a function of CaCO_3_ added (g/l) with the *pdc1Δ::LhLDH pdc2Δ::LhLDH* transformant (**A**) in YP+10% (w/v) and (**B**) in YNB+10% glucose medium after 144 h incubation at 30**°**C.

In the previous experiments less than 14 g/l lactate was produced in medium with no pH buffering (final pH 3.3). The *pdc1Δ::LhLDH pdc2Δ::LhLDH* strain, was also grown in YP-10% glucose medium supplemented with calcium carbonate (CaCO_3_) concentrations from 5 to 30 g/l as a neutralizing agent to control the pH and to determine the relationship between free lactic acid and total lactate production. The final pH in the cultures was between pH 3.5 and 4 (Figure 6A), around the pKa of lactic acid (pH 3.8). The total lactate concentration (24 to 66 g/l), lactate yield on glucose, and final pH increased with increasing CaCO_3_ concentration, but the concentration of free lactic acid varied relatively little between the conditions and was maximal, 19 g/l, at low CaCO_3_ concentrations (Figure 6A). The proportion of free lactic acid in the total lactate decreased with increasing CaCO_3_ concentration and final pH from ~80% at final pH 3.5 to ~20% at final pH 4. A similar correlation between CaCO_3_ and lactate concentrations was also observed on YNB-10% glucose medium (Figure 6 B) but the lactate concentration was 10 g/l higher in rich YP medium than in YNB-medium at each CaCO_3_ concentration between 5 and 25 g/l (Figure 6). The pH in YP and YNB media were similar at each CaCO_3_ concentration although the lactate concentrations differed.

### Lactate production in cultivations containing a non-limiting concentration of CaCO_3_

*LhLDH, BmLDH* and *RoLDH* strains, with and without *PDC* modifications, were studied for lactate production on YNB-10% glucose minimal medium using a two-stage cultivation protocol with separate biomass generation and lactate production phases. The production phase was inoculated with a biomass concentration of 5 g/l cell dry weight and the pH was maintained above 5.7 by addition of CaCO_3_.

**Figure 7:**
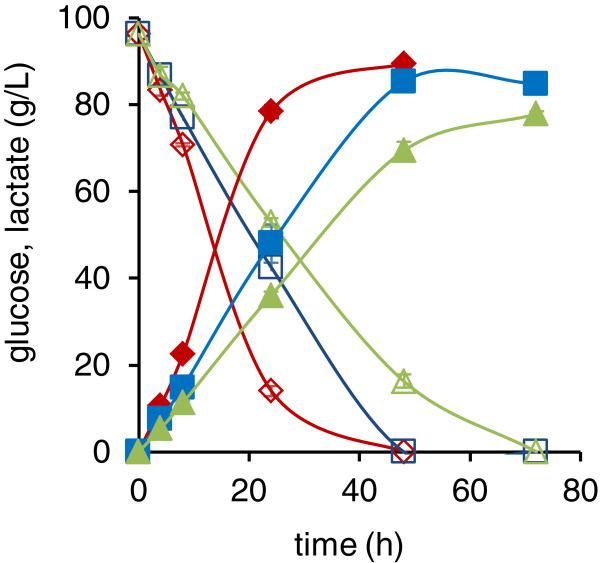
**Lactate production in the presence of a non-limiting concentration of CaCO**_**3**_**.** Lactate and glucose concentrations in YNB +10% (w/v) glucose + CaCO_3_ medium by the *pdc1Δ::LhLDH pdc2Δ* (♦), *pdc1Δ::BmLDH pdc2Δ* (■), and *pdc1Δ::RoLDH pdc2Δ* ▲strains. Data are means (n=2).

The *LhLDH, BmLDH* and *RoLDH* strains produced up to 93, 88 and 78 g/l lactate, respectively (Table 1). Glucose consumption and lactate production for representative *pdc1Δ::LDH pdc2Δ* are shown in Figure 7. The lactate production rate during the first 24 hours was the highest with the *pdc1Δ::LhLDH pdc2Δ* strain (3.3 g/l/h) followed by the *pdc1Δ::BmLDH pdc2Δ* (2.0 g/l/h) and *pdc1Δ::RoLDH pdc2Δ* (1.5 g/l/h). A visible calcium lactate precipitate was formed in some cultivations of the *LhLDH* or *BmLDH* strain, but was never formed in the *RoLDH* cultivations. The *LhLDH* strains produced on average 5 g/l more lactate than the corresponding *BmLDH* strains when the *PDC1* gene or both *PDC* genes were deleted, but the difference was not statistically significant. The optical purity of the lactate was high, since the concentration of d-lactate was below 0.6 g/l (determined enzymatically from samples containing the maximum lactate concentration). Thus more than 99% of the total lactate was l-lactate.

**Table 1:** **Extracellular metabolites produced in the presence of CaCO**_**3**_

**Strain**	**Lactate (g/l)**	**Yield of lactate (g/g)**	**Ethanol (g/l)**	**Pyruvate (g/l)**
*x::BmLDH*	85 ± 1.7	0.89 ± 0.02	≤ 0.3	≤ 0.2
*pdc1∆::BmLDH*	88 ± 1.4	0.90 ± 0.01	1.3 ± 0.4	≤ 0.5
*pdc2∆::BmLDH*	84 ± 2.6	0.87 ± 0.02	1.2 ± 0.6	≤ 0.6
*pdc1∆::BmLDH pdc2∆*	84 ± 2.9	0.85 ± 0.03	n.d.	2.1 ± 0.3
*pdc1∆::BmLDH pdc2∆::BmLDH*	81 ± 3.2	0.80 ± 0.03	n.d.	0.79 ± 0.02
*x::LhLDH*	83 ± 1.7	0.85 ± 0.02	≤ 0.3	n.a.
*pdc1∆::LhLDH*	93 ±0.8	0.95 ± 0.01	1.3 ± 0.3	≤ 0.5
*pdc1∆::LhLDH pdc2∆*	92 ± 1.6	0.94 ± 0.02	n.d.	0.72 ± 0.13
*pdc1∆::LhLDH pdc2∆::LhLDH*	86 ±1.3	0.88 ± 0.02	n.d.	0.39 ± 0.08
*x::RoLDH*	78 ± 1.5	0.81 ± 0.02	3.2 ± 0.2	n.a.
*pdc1∆::RoLDH*	75 ± 0.6	0.77 ± 0.02	7.0 ± 1.2	< 0.5
*pdc1∆::RoLDH pdc2∆*	78 ± 0.2	0.81 ± 0.01	n.d.	3.2 ± 0.3
wild type *C. sonorensis*	n.d.	n.d.	17 ± 5.3	n.a.

Maximum concentrations of lactate, ethanol and pyruvate (g/l) and lactate yield on glucose (g/g) produced in YNB-10% (w/v) glucose minimal medium containing non-limiting concentration of CaCO_3_. Results are from 6 experiments, each with 4–6 strains. Data are means ± SEM (n= 3–10). n.a. not analyzed. n.d. not detected.

The ethanol concentrations were small even for the strains with intact *PDC1* and/or *PDC2* (Table 1) in YNB-10% glucose medium with CaCO_3_. The *RoLDH* strains with an intact *PDC2* produced significantly (p < 0.05) more ethanol than the *LhLDH* or *BmLDH* strains with an intact *PDC2* (Table 1).

Pyruvate concentrations produced by the *pdc1Δ::LDH pdc2Δ* strains were significantly different (p < 0.05) for *LhLDH, BmLDH,* and *RoLDH* strains. The pyruvate concentration was also lower for the strains containing two copies of *BmLDH* or *LhLDH* than for the corresponding strains with a single *LDH* gene copy (Table 1), as in the non-buffered cultivations (see above).

In the absence of both *PDC* genes no ethanol was produced. As the by-product concentrations for all strains were extremely small relative to lactate concentrations, and decrease in ethanol concentration was accompanied with an increase in pyruvate concentration, double *PDC* deletions did not result in an increase in lactate concentration or yield on glucose.

### Intracellular lactate and pyruvate concentrations

**Figure 8:**
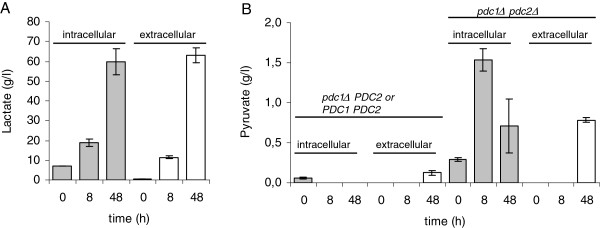
**Intracellular and extracellular lactate and pyruvate.** (**A**) Concentrations of intra- (grey bars) and extracellular (white bars) lactate in strains expressing *BmLDH* in cultures containing non-limiting concentration of CaCO_3_. Data are means ± SEM (n=7). (**B**) Concentrations of intra- (grey bars) and extracellular (white bars) pyruvate in strains containing an intact *PDC2*, and in *pdc1Δ pdc2Δ* strains, all expressing *BmLDH*. Data are means ± SEM (n=3-4). Pyruvate concentrations below 0.03 g/l were not detectable.

Intracellular and extracellular lactate concentrations were correlated in *PDC* positive *BmLDH* strains cultivated in CaCO_3_-buffered minimal media. Cells in CaCO_3_-buffered medium had more intracellular lactate compared to extracellular lactate at the beginning of the cultivations (0 and 8 hours). At the end of cultivation (48 hours) the intra- and extracellular lactate concentrations were similar (up to 80 g/l) (Figure 8A).

The intracellular pyruvate concentration was higher in *pdc1Δ pdc2Δ* strains than in strains containing an intact *PDC2* (*pdc1Δ PDC2* or *PDC1 PDC2*), as was the extracellular concentration (Figure 8B).

## Discussion

New molecular tools have enabled genetic engineering of *C. sonorensis* for the first time. The antibiotic marker gene G418^R^ and the non-antibiotic marker *MEL5* were expressed under the control of endogenous *PGK1* or *TDH1* promoters, and the *PGK1* promoter was additionally used to express three different *LDH* genes. Targeted integration into *PDC1* and *PDC2* through homologous recombination was common but not as frequent as integration into non-homologous sites in the genome. In addition to *PDC* loci, homologous integration into the *PGK1* locus occurred in some transformants when the marker gene was located between two identical *PGK1* promoter copies in the construct, *e.g.* in pMI257 or pMI265.

*C. sonorensis* has two non-allelic *PDC* genes, *PDC1* and *PDC2*, both of which contribute to ethanol production. The *PDC2* gene encodes the major isoenzyme. *PDC2* deletion resulted in a decrease in growth, glucose utilization and ethanol production rates, and in an increase in pyruvate levels. In contrast, the *PDC1* deleted strain did not noticeably differ from the parent strain in respect to these parameters, and 85% of PDC activity was retained*.* Both intra- and extracellular pyruvate concentrations were significantly increased in the *C. sonorensis pdc1Δ pdc2Δ* strain, compared with strains containing an intact *PDC2* gene. The expression of any of the three *LDH* genes in the *C. sonorensis pdc1Δ pdc2Δ* strain background provided an alternative route for pyruvate metabolism and NAD^+^ regeneration, and was accompanied with a significant decrease in pyruvate accumulation, particularly in the *LhLDH* strain. *LDH* expression in a *pdc1Δ pdc2Δ* strain also enhanced glucose consumption in non-buffered medium, with LhLDH having a greater positive effect than the other two LDHs had. Even so, glucose consumption by a *pdc1Δ::LhLDH pdc2Δ* strain was slow relative to the ethanol producing *LDH* strains in non-buffered medium, as also observed in *S. cerevisiae*[2].

*C. sonorensis* strains expressing *LDH* from *L. helveticus*, *B. megaterium* or *R. oryzae*, showed characteristic differences in the conversion of glucose to lactate and by-products, demonstrating that the properties of the LDH enzyme have a fundamental impact on carbon distribution at the pyruvate branch point. In general, the concentration and yield on glucose of the by-products ethanol (PDC+) or pyruvate (*pdc1∆ pdc2∆*) were the highest with the *RoLDH s*trains and the lowest with the *LhLDH* strains. The efficiency of lactate production corresponded to the LDH enzyme activity, with the *LhLDH* strain having the highest, the *BmLDH* strain intermediate, and the *RoLDH* strain the lowest *in vitro* activity in single-copy *LDH* strains. *S. cerevisiae* strains expressing a LDH from *Lactobacilli* had higher LDH activity than a *BmLDH* expressing strain [15]*.* Data on the properties of the LDH enzymes used are limited, but significant differences have been reported [15,19,20]. The kinetic properties such as the pH optimum, affinity for pyruvate and the cofactor NADH, inhibition by a high substrate or product concentration, and the equilibrium of the reaction would determine the effectiveness of each enzyme *in vivo*. Indeed, the *C. sonorensis* strains with different LDH enzymes produced different final extracellular lactate and pyruvate concentrations, which were shown to correlate with intracellular concentrations.

Concentrations of intracellular lactate have not been reported for *LDH* expressing yeasts to date. The present initial work found that lactic acid producing *C. sonorensis* cells harvested from un-buffered cultures contained a significant intracellular lactic acid concentration. This may interfere with multiple cellular functions, but in spite of this, the cells were able to excrete lactate. It has been proposed that lactate export is energy dependent and uses ATP in *S. cerevisiae*[21]. The lactate and acetate transporters *JEN1* and *ADY2* that are known to import lactate are also involved in lactate excretion, but another presently unknown lactate export mechanism also exists in *S. cerevisiae*[22,23].

Lactic acid accumulation decreases the pH of the culture medium leading to an increase in the proportion of undissociated lactic acid in the medium. At pH 4, a fraction of the lactic acid will be undissociated. Undissociated acid is believed to re-enter the cell also via passive diffusion. In the cytosol, at neutral pH, it will dissociate to form the lactate anion and proton, thus increasing the ATP demand for lactate export [21]. Cytosolic acidification caused by lactic acid may eventually result in cell death [24].

Lactic acid accumulation causes also weak acid stress to the cells. Cells exposed to weak acids adapt to some extent, for instance by up-regulating excretion of the acid, by blocking re-entry, or by metabolizing the acid [25]. Different yeast species may use different strategies to maintain cellular pH and ion homeostasis. The present data showed that lactate concentration in the culture medium decreased in prolonged cultivations indicating that the cells consumed lactate (see e.g. Figure 2).

An optimal lactic acid production host should tolerate acidic conditions and produce a high concentration of undissociated lactic acid in order to reduce the need for neutralizing chemicals, and ethanol production should be eliminated. As shown in Figure 6, the media composition (YP or YNB) and the amount of CaCO_3_ determine how much lactic acid a strain can produce. The medium composition is an important consideration in production process because of cost, downstream processing, and product quality, which was why minimal medium (YNB) was mainly used in the present work. It is evident that differences in the culture conditions used by different groups complicate fair comparison between the species. Not surprisingly, the highest total lactate production levels by yeasts have been obtained at higher pH using non-limiting concentration of neutralizing chemicals.

When benchmarked against published results where media and operating conditions are disclosed, this *C. sonorensis* strain compares favorably. Representative results for lactate producing yeast strains, which produce little or no ethanol, at neutral pH have been reported for *C. boidinii* that produced 86 g/l lactic acid in the presence of non-limiting CaCO_3_ and final pH 6.15 [8], and *C. sonorensis* that produced 92 g/l lactate at 0.94 g/g yield on glucose in less than 48 h. *S. cerevisiae pdc1∆ pdc5∆* strain produced 82 g/l lactate at 0.82 g/g yield in YP-10% glucose in the presence of 30 g/l CaCO_3_, but the pH was not reported [2]. *C. utilis* produced 103 g/l lactic acid in YP medium containing 109 g/l glucose (0.95 g/g yield on glucose) and 45 g/l CaCO_3_ in in 33 h, with final pH of 4 [9]. *S. cerevisiae* wine yeast produced 40 g/l lactate [3], and diploid *S. cerevisiae* produced 50 g/l lactate below pH 4 [14]. *C. boidinii* produced 50 g/l lactic acid in YP-10 g/l glucose medium containing 30 g/l CaCO_3_. In comparison, *C. sonorensis LhLDH* strains produced in YP medium 66 g/l lactate (0.73 g/g yield) with 30 g/L of CaCO_3_ and final pH 4.0. The LDH and PDC modifications are the necessary basis for further yeast development towards an industrial lactic acid process.

## Conclusions

We developed an organism without previous record of genetic engineering to efficiently produce L-lactic. Genetic modification of *C. sonorensis* opens the possibility to exploit this novel host organism in the production of useful biochemicals. The frequent occurrence of both targeted and non-homologous integration into the genome gives flexibility to strain design and construction. Both PDC1 and PDC2 enzymes contributed to ethanol production, but *PDC2* encodes the main isoenzyme. The possibility to generate knock out strains allowed us to demonstrate the significance of each of the *PDC* genes in the context of lactic acid production in *C. sonorensis*. Unexpectedly, *LDH* strains with intact *PDC* genes produced very little ethanol and as much lactate as the *PDC* deleted strains in the presence of CaCO_3_, although the same strains produced more ethanol than lactate in non-buffered conditions. This indicated that not only the genotype but also the culture conditions had a large influence on carbon distribution between ethanol and lactate.

The Cargill commercial implementation of a yeast for lactic acid production has demonstrated the high potential of yeasts as hosts for organic acid production [26]. This present work showed that glucose could be converted to highly pure l-lactate at an excellent yield by *C. sonorensis* expressing a *LDH* in minimal medium in the presence of CaCO_3_. The purity of the product i.e. taking into account the formation of by-products ethanol and pyruvate, and to some extent the concentration of lactate differed between strains expressing different *LDH* genes. The lactate production parameters (concentration, yield, production rate) observed with *C. sonorensis* strains expressing *LhLDH* compare favorably with other lactic acid producing yeasts*,* illustrating that *Candida* yeasts have high potential as lactic acid production hosts. Among the *LDH* genes studied, *LhLDH* was the most suitable one to produce lactic acid with *C. sonorensis* in the conditions studied. Thus, the choice of the LDH is an important consideration in the development of improved production hosts.

## Methods

### Microbial strains

*E. coli* strains DH5α (Gibco BRL, Gaithersburg, MD) and XL-1 Blue (Stratagene, La Jolla, CA) were used as hosts for cloning and plasmid propagation. *C. sonorensis* ATCC32109 (American Type Culture Collection), was used throughout the study and was the parental strain of the transformants generated in this work.

### Media and cultivation conditions

*C. sonorensis* was maintained on agar solidified 1% (w/v) yeast extract – 2% (w/v) Bacto peptone – 2% (w/v) glucose (YPD) medium supplemented with 200 mg/l geneticin (G-418 sulfate; Invitrogen, Carlsbad, CA, USA) or 40 mg/l 5-bromo-4-chloro-3-indolyl-α-D-galactopyranoside (X-α-Gal; ICN Biochemicals, Aurora, OH, USA), as appropriate. Test tube cultivations were carried out in 5 ml 1% (w/v) yeast extract – 2% (w/v) peptone medium (YP) containing 5% (w/v) glucose for initial tests for lactate and ethanol production and were incubated at 250 rpm.

Non-buffered cultivations in YP - 5% (w/v) glucose were inoculated to an optical density (OD_600_) of 0.2 with cells grown on YPD agar. In some experiments the YP medium contained 10% (w/v) glucose and 5 to 30 g/l of calcium carbonate (CaCO_3_) for pH control.

For two-stage cultivations, the biomass was grown in yeast nitrogen base medium (YNB w/o amino acids; Difco, Sparks, MD) supplemented with 5% glucose and buffered to pH 5.5 with 0.5 M 2-[N-Morpholino]ethanesulfonic acid (MES). After overnight cultivation at 30°C and 250 rpm, cells were harvested by centrifugation and transferred into YNB medium supplemented with 10% (w/v) glucose, to give an initial cell density of OD_600_ ~ 15 corresponding to approximately 5 g/l cell dry weight. 80 g/l of calcium carbonate was added for pH control in some of the cultures.

Cultures were incubated at 30°C with 100 rpm shaking in 250 ml Erlenmeyer flasks containing 50 ml medium.

### DNA manipulations

Plasmid DNA was isolated using Qiagen kits (Qiagen Corp, Chatsworth, CA, USA). Recombinant DNA work was carried out using conventional techniques [27]. Oligonucleotides were purchased from Sigma-Genosys (Little Chalfont, UK). PCR was performed using Dynazyme EXT polymerase (Finnzymes, Espoo, Finland) with an initial incubation for 3 min at 94°C, followed by 29 cycles of 45 sec at 94°C, 45 sec at 55°C, 2 min at 72°C, with a final incubation for 10 min at 72°C.

### Isolation of *PGK1, TDH1, PDC1* and *PDC2* genes from *C. sonorensis*

Yeast DNA was isolated by phenol extraction from cells broken with glass beads [28]. The genomic library of *C. sonorensis* ATCC32109 was prepared using partially *Sau*3A digested size fractionated genomic DNA that was cloned into the *Bam*HI digested lambda DASH™ vector (Stratagene, La Jolla, CA, USA) as described previously [29]. The library was screened by colony/plaque hybridization. *C. albicans PGK1*, amplified by PCR from genomic DNA with primers 5092 and 5091 was used as a probe to isolate the *C. sonorensis* gene for 3-phosphoglycerate kinase (*PGK*), and *S. cerevisiae TDH1,* amplified with primers 4125 and 4126 (Table 2) was used as a probe to isolate the gene for glyceraldehyde-3-phosphate dehydrogenase (GAPDH). Fragments of *PDC1* and *PDC2,* were amplified from genomic DNA of *C. sonorensis* with primers 5116 and 5118 (Table 2) which were designed from conserved regions in the known pyruvate decarboxylase amino acid sequences, WAGNANELNA and DFNTGSFSY, of *P. stipitis PDC1* (U75310) and *PDC2* (U75311), *S. cerevisiae PDC1* (X04675), and *C. albicans PDC11* and *PDC12* (sequence data for *C. albicans* was obtained from the Candida Genome Database website at http://www.candidagenome.org/). The *C. sonorensis PDC1* and *PDC2* fragments obtained with primers 5116 and 5118 (Table 2) were used as probes for the isolation of the corresponding genes from a genomic library. The identity of the purified genomic clones was verified by DNA sequencing.

**Table 2:** Oligonucleotides used in this work

**Name**	**Sequence**	**Description**
4125	5′-tgtcatcactgctccatctt-3′	*S. cerevisiae TDH1* gene
4126	5′-ttaagccttggcaacatatt-3′	*S. cerevisiae TDH1* gene
5092	5′-gcgatctcgaggtcctagaatatgtatactaatttgc-3′	*C. albicans PGK1* ORF (acc. U25180)
5091	5′-cgcgaattcccatggttagtttttgttggaaagagcaac-3′	*C. albicans PGK1* ORF (acc. U25180)
5423	5′-gcgatctcgagaaagaaacgacccatccaagtgatg-3′	*CsPGK1* promoter −1500
5439	5′-tggactagtacatgcatgcggtgagaaagtagaaagcaaacattgtatatagtcttttctattattag-3′	*CsPGK1* promoter*-MEL5* fusion
5441	5′-gcgatctcgagaaaatgttattataacactacac-3′	*CsTDH1* promoter −*600*
5440	5′-tggactagtacatgcatgcggtgagaaagtagaaagcaaacattttgtttgatttgtttgttttgtttttgtttg-3′	*CsTDH1* promoter *MEL5* fusion
5427	5′-acttggccatggtatatagtcttttctattattag-3′	*CsPGK1* promoter *–LhLDH* fusion
LhLDH1	5′-atggcaagagaggaaaaacctcgtaaag-3′	*LhLDH* probe (fwd)
LhLDH2	5′-ccacgaagagtcattgacgaaccttaa-3′	*LhLDH* probe (rev)
BmLDH1	5′-ccaacaaaaccagttccgataacg-3′	*BmLDH* probe (fwd)
ScerGal10t	5′-ccggactagttggtacagagaacttgtaaacaattcgg-3′	*BmLDH* probe (rev)
RoLDHA1	5′-ctagctcagaacaatggtattacactcaaaggtcgccatcg-3′	*RoLDH* probe (fwd)
RoLDHA2	5′-cgcggatccgaattctcaacagctacttttagaaaaggaag-3′	*RoLDH* probe (rev)
5116	5′-ccggaattcgatatctgggcwggkaatgccaaygarttraatgc-3′	*PDC1* and *PDC2* probes (fwd)
5118	5′-cgcggattcaggcctcagtangaraawgaaccngtrttraartc-3′	*PDC1* and *PDC2* probes (rev)
G418-5′	5′-ctagtctagaacaatgagccatattcaacgggaaacg-3′	*G418*^R^ probe (fwd)
G418-3′	5′-cgcggatccgaattcttagaaaaactcatcgagcatcaaatg-3′	*G418*^R^ probe (rev)
Cs1	5′-ctagtctagatttgtttgatttgtttgttttgtttttgtttg-3′	*C. sonorensis TDH1* promoter
Cs2	5′-ctagtctagatgtatatagtcttttctattattag-3′	*C. sonorensis PGK1* promoter
Cs5	5′-ggcccgcggccgctacaagtgattcattcattcact-3′	*C. sonorensis PDC1* 5′ flank
Cs6	5′-ccctgggcccctcgaggatgatttagcaagaataaattaaaatgg-3′	*C. sonorensis PDC1* 5′ flank
Cs7	5′-gggactagtggatccttgaagtgagtcagccataaggacttaaattcacc-3′	*C. sonorensis PDC1* 3′ flank
Cs8	5′-aaggccttgtcgacgcggccgcttggttagaaaaggttgtgccaatttagcc-3′	*C. sonorensis PDC1* 3′ flank
Cs26	5′-gggacgggcccgcggccgcttacagcagcaaacaagtgatgcc-3′	*C. sonorensis PDC2* 5′ flank
Cs27	5′-ccctgggcccctcgagtttgatttatttgctttgtaaagagaa-3′	*C. sonorensis PDC2* 5′ flank
Cs29	5′-tggactagttagatagcaattcttacttgaaaaattaattgaagcattacc-3′	*C. sonorensis PDC2* 3′ flank
Cs30	5′-ggcccgcggccgctaaatataattatcgcttagttattaaaatgg-3′	*C. sonorensis PDC2* 3′ flank

### Southern and colony/plaque hybridizations

Southern blots were prepared using conventional techniques and hybridized with probes labeled with [α-^32^P]dATP or [α-^32^P]dCTP (Amersham Pharmacia, Little Chalfont, UK) or with digoxigenin-11-dUTP (Roche, Mannheim, Germany). The presence and copy number of the *LDH* gene was verified by Southern analysis of *Hin*dIII digested yeast DNA using the corresponding *LDH* gene as the probe (see Table 2 for probe PCR primers). The *PDC1*- or *PDC2* deletions were verified by Southern analyses by the absence of *PDC1*- or *PDC2*-specific hybridization signals and the appearance of transformation marker–specific signals of appropriate size. *PDC1* or *PDC2* probes correspond to nucleotides in the deleted area and were amplified by PCR. Radioactive hybridization signals were detected by scanning exposed storage phosphor screens using the Typhoon 8600 variable mode imager (Molecular Dynamics, Sunnyvale, CA). Non-radioactive signals were detected colorimetrically with NBT and BCIP (Promega, Madison, WI).

### Plasmid construction

Plasmids were constructed using conventional techniques [27]. Oligonucleotides were purchased from Sigma Genosys (Haverhill, UK). Dynazyme EXT or Phusion™ polymerase (Finnzymes, Espoo, Finland) were used for routine PCR amplification. The *S. cerevisiae MEL5* gene (Genbank accession number Z37511) [30,31] was obtained as a 2.2 kb *Eco*RI-*Spe*I fragment from plasmid pMEL5-39 and ligated to *Eco*RI-*Spe*I cut pBluescript II KS(−) (Stratagene). The 1.5 kb *C. sonorensis PGK1* promoter was amplified with primers 5423 and 5439 (Table 2) from a *PGK1* lambda clone isolated from the genomic library and inserted upstream of the *MEL5* ORF using *Sph*I and *Xho*I enzymes resulting in pMI234 (Table 3). A similar strategy was used to construct pMI238 (Table 3) that contains the 0.6 kb *C. sonorensis* GAPDH (*TDH1*) promoter, amplified with primers 5441 and 5440, upstream of *MEL5.* The 1.3 kb *Nco*I-*Bam*HI fragment of pVR1 (V. Rajgarhia, NatureWorks LLC) containing the *LhLDH* gene and the *S. cerevisiae CYC1* terminator was ligated to the 1.5 kb *C. sonorensis PGK1* promoter, which was amplified with primers 5423 and 5427, and alternatively, to the 0.6 kb *C. sonorensis TDH1* promoter amplified with primers 5441 and 5440. The *LhLDH* expression cassette obtained as a 3.4 kb *Avr*II-*Nhe*I fragment, was inserted into *Spe*I digested pMI234 resulting in pMI246 (Table 3). pMI246 was further modified in two steps for the replacement of *PDC1.* The *C. sonorensis PDC1* 3′ homology region inserted downstream of the *LhLDH* expression cassette, was amplified from genomic DNA using primers Cs7 and Cs8 (Table 2), digested with *Bam*HI and *Not*I and ligated with *Bam*HI-*Not*I digested pMI246 (8.9 kb), generating pMI256 (Table 3). The *PDC1* 5′ homology region, inserted upstream of the *MEL5* marker cassette, was amplified with primers Cs5 and Cs6 (Table 2), digested with *Apa*I, and ligated with the 9.8 kb pMI256 linearised with *Apa*I, generating pMI257 that contains *C. sonorensis PDC1* 5′ homology region (0.8 kb), *C. sonorensis PGK1* promoter*, S. cerevisiae MEL5, C. sonorensis PGK1* promoter*, L. helveticus ldhL* (*LhLDH*) [19]*, S. cerevisiae CYC1* terminator and *C. sonorensis PDC1* 3′ homology region (0.9 kb), in that order (Table 3). It was modified by replacing the *LhLDH* with *B. megaterium ldh* (*BmLDH;* GenBank accession no. M22305) in pMI265 or the *R. oryzae ldhA* (*RoLDH*, GenBank accession AF226154) [20] in pMI266 (Table 3). A control vector lacking *ldhL*, pMI267 (Table 3), was constructed by removing the *ldhL* from pMI257 with *Nco*I and *Bam*HI digestion, filling the overhangs in, and circularizing the 9.2 kb fragment.

**Table 3:** **Plasmids for *C. sonorensis *transformations constructed in this work**

**Plasmid**	**Relevant content**
pMI234	*CsP*_*PGK1*_*-ScMEL5*
pMI238	*CsP*_*TDH1-*_*ScMEL5*
pMI246	*CsP*_*PGK1*_*-ScMEL5-ScT*_*MEL5*_*- CsP*_*PGK1*_*-LhLDH-ScT*_*CYC1*_
pMI247	*CsP*_*GPD1*_*-ScMEL5-ScT*_*MEL5*_*- CsP*_*PGK1*_*-LhLDH-ScT*_*CYC1*_
pMI257	*CsPDC1 5′ - CsP*_*PGK1*_*-ScMEL5-ScT*_*MEL5*_*- CsP*_*PGK1*_*-LhLDH-ScT*_*CYC1*_*- CsPDC1 3′*
pMI265	*CsPDC1 5′ - CsP*_*PGK1*_*-ScMEL5-ScT*_*MEL5*_*- CsP*_*PGK1*_*-BmLDH-CsPDC1 3′*
pMI266	*CsPDC1 5′ - CsP*_*PGK1*_*-ScMEL5-ScT*_*MEL5*_*- CsP*_*PGK1*_*-RoLDH-CsPDC1 3′*
pMI267	*CsPDC1 5′ - CsP*_*PGK1*_*-ScMEL5-ScT*_*MEL5*_*- CsP*_*PGK1*_*-CsPDC1 3′*
pMI268	*CsP*_*PGK1*_*1-G418*^R^*-ScT*_*GAL10*_
pMI269	*CsP*_*GPD1*_*-G418*^R^*-ScT*_*GAL10*_
pMI278	*CsP*_*GPD1*_*-G418*^R^*-ScT*_*MEL5*_*- CsP*_*PGK1*_*-BmLDH-ScT*_*GAL10*_
pMI279	*CsPDC2 5′* - *CsP*_*GPD1*_*-G418*^R^*-ScT*_*MEL5*_*- CsP*_*PGK1*_*-BmLDH-ScT*_*GAL10*_
pMI286	*CsPDC2 5′ - CsP*_*GPD1*_*-G418*^R^*-ScT*_*MEL5*_*- CsP*_*PGK1*_*-BmLDH-ScT*_*GAL10*_*- CsPDC2 3′*
pMI287	*CsPDC2 5′- CsP*_*GPD1*_*-G418*^R^*-ScT*_*MEL5*_*- CsP*_*PGK1-*_*CsPDC2 3′*
pMI288	*CsPDC2 5′- CsP*_*GPD1*_*- G418*^R^*-ScT*_*MEL5*_*- CsP*_*PGK1*_*-LhLDH-ScT*_*CYC1*_*- CsPDC2 3′*

The *G418*^R^ gene was amplified with primers G418-5′ and G418-3′ (Table 2) from pPIC9K (Invitrogen), the 0.8 kb PCR product was digested with *Bam*HI and *Xba*I and ligated to the 4.2 kb *Bam*HI-*Xba*I fragment of pNC101 (E. Jarvis, NREL, Golden, CO, USA) between *S. cerevisiae PGK1* promoter and terminator generating pMI260. The promoter was replaced by the *C. sonorensis TDH1* promoter, which was amplified from pMI238 with primers 5441 and Cs1, made blunt ended, digested with *Xba*I, and ligated with the 4.2 kb *Pst*I (blunt)-*Xba*I fragment of pMI260 to generate pMI269 (Table 3). pMI268 (Table 3), that contains the *C. sonorensis PGK1* promoter amplified with primers 5423 and Cs2 (Table 2) from pMI234, upstream of *G418*^R^, was constructed similarly as pMI269.

Plasmids for replacement of the *PDC2* locus containing *C. sonorensis PDC2* 5′ homology region (0.8 kb), *C. sonorensis GPD1* promoter*, E. coli G418*^R^, *S. cerevisiae MEL5* terminator, *C. sonorensis PGK1* promoter, one of the *LDH* genes, *S. cerevisiae GAL10* terminator and *C. sonorensis PDC2* 3′ homology region (0.9 kb), were prepared as follows. The *BmLDH* from pMI265 and the *G418*^R^ expression cassettes were joined to form pMI278 (Table 3). The region upstream of *PDC2* ORF was amplified by PCR using the primers Cs26 and Cs27 (Table 2), and the genomic copy of the *C. sonorensis PDC2* as the template (GenBank accession number AM420320), and the PCR product was inserted upstream of the LDH expression cassette resulting in plasmid pMI279. Then the 0.9 kb *PDC2* 3′ homology region amplified by PCR as above using primers Cs29 and Cs30 (Table 2) was added to form pMI286. *BmLDH* in pMI286 was replaced by *LhLDH* resulting in pMI288. pMI287 was constructed by removing *BmLDH* from pMI286.

### Transformation of *C. sonorensis*

All plasmids were digested with restriction enzymes prior to transformation to facilitate integration into the genome, unless otherwise stated. *C. sonorensis* was transformed using the lithium acetate method [32,33]. After 3 hours incubation in liquid YPD medium, cells were spread onto agar-solidified YPD medium containing 200 μg/ml G-418 sulfate, or 40 μg/ml X-α-Gal (ICN Biochemicals, Aurora, OH, USA), a chromogenic substrate of α-galactosidase. Cells transformed with *Not*I digested pMI257 (*LhLDH*), pMI265 (*BmLDH*), pMI266 (*RoLDH*), or pMI267 (no *LDH*) were selected for melibiase activity, and with pMI286 (*BmLDH*), pMI287 (no *LDH*) or pMI288 (*LhLDH*) were selected for G418 resistance. The *PDC2* gene was replaced by the *G418*^R^ gene in the *pdc1* deleted, melibiase positive *C. sonorensis* transformants containing the *LhLDH*, *BmLDH*, *RoLDH*, or no *LDH* by transformation with pMI287 (no *LDH*). In addition, *PDC2* was replaced by the *G418*^R^ and *LDH* genes by introducing a second copy of *LhLDH* or *BmLDH* into strains containing the *LhLDH* or *BmLDH* gene, respectively, integrated in the *PDC1* locus. Putative *PDC2* deletants were screened for decreased ethanol production. Replacements of *PDC1* or *PDC2* genes and the presence of *LDH* were verified by Southern analyses. Strains constructed in this work are listed in Table 4.

**Table 4:** ***C. sonorensis *ATCC32109 derived strains constructed and studied in this work**

**Description**	**Transformed with plasmid(s)**
*pdc1∆*	pMI267
*pdc2∆*	pMI287
*pdc1∆ pdc2∆*	pMI267, pMI287
*pdc1∆::BmLDH*	pMI265
*pdc2∆::BmLDH*	pMI286
*pdc1∆::BmLDH pdc2∆*	pMI265, pMI287
*x::BmLDH*	pMI265
*x::BmLDH y::BmLDH*	pMI265
*x::LhLDH*	pMI246
*x::LhLDH*	pMI247
*x::LhLDH y::LhLDH*	pMI257
*x::LhLDH-LhLDH-LhLDH*	pMI247
*pdc1∆::LhLDH*	pMI257
*pdc1∆::RoLDH*	pMI266
*pdc1∆::LhLDH pdc2∆*	pMI257, pMI287
*pdc1∆::RoLDH pdc2∆*	pMI266, pMI287
*pdc1∆::LhLDH pdc2∆::LhLDH*	pMI257, pMI288
*pdc1∆::BmLDH pdc2∆::BmLDH*	pMI265, pMI286

The heterologous *LhLDH*, *BmLDH*, and *RoLDH* genes were expressed under the control of the *C. sonorensis PGK1* promoter; the *MEL5* and *G418*^R^ marker genes were expressed under the *C. sonorensis GPD1* or *PGK1* promoter (not indicated in the table). *x::* and *y::* indicate that the site of integration is not known. Two consecutive transformations were made to construct a strain where two plasmids are listed.

### PDC and LDH enzyme activity measurements

Enzyme activities were measured from freshly prepared cell extracts. Cells from 5 ml samples were harvested by centrifugation, washed with 1 ml of ice-cold 10 mM K_2_HPO_4_/ KH_2_PO_4_, pH 7.5, 2 mM EDTA, then with 1 ml of homogenization buffer [(100 mM KH_2_PO_4_/ K_2_HPO_4_, pH 7.5, 2 mM MgCl_2_, 1 mM DTT containing protease inhibitors (Complete Mini, EDTA free, Roche)], resuspended in 0.75 ml of homogenization buffer and homogenized with 0.75 ml glass beads using a Mini Bead Beater (BioSpec Products, Bartlesville, OK) for 4 × 30 seconds. Samples were centrifuged at 14 000 rpm for 30 min at 4°C. PDC activity was determined spectrophotometrically (A_340_) with a Cobas Mira automated analyser at 30°C in 40 mM imidazole-HCl (pH 6.5) containing 0.2 mM NADH, 50 mM MgCl_2_, 0.2 mM thiamine pyrophosphate, 90 units alcohol dehydrogenase, and 50 mM pyruvate. LDH enzyme activity in the supernatant was determined spectrophotometrically (A_340_) with a Cobas Mira automated analyzer at 30°C in 50 mM sodium acetate (pH 5.2) and in 50 mM imidazole-HCl (pH 6.5) buffer, each containing 0.4 mM NADH, 5 mM fructose-1, 6-diphosphate (FBP) and 2 mM pyruvate. *R. oryzae* LDH activity was measured in the presence and in the absence of FBP at pH 6.5. The activities are expressed in units per milligram protein (U/mg). 1 U was defined as the amount of enzyme required to reduce 1 μmol of substrates per min. Protein concentrations were measured using a protein assay reagent (Bio-Rad 500–0006) and bovine serum albumin (Sigma) as the protein standard.

### Analytical methods

The culture supernatants were analyzed by HPLC for lactic acid, glucose, pyruvic acid, acetic acid, glycerol and ethanol using a Waters 2690 Separation Module and Waters System Interphase Module liquid chromatography coupled with a Waters 2414 differential refractometer and a Waters 2487 dual λ absorbance detector (Waters, Milford, MA). A Fast Juice Column (50 mm × 7.8 mm, Phenomenex, Torrance, CA) and a Fast Acid Analysis Column (100 mm × 7.8 mm, Bio-Rad, Hercules, CA) or, alternatively, a Fast Acid Analysis Column (100 mm × 7,8 mm, Bio-Rad) and an Aminex HPX-87H Organic Acid Analysis Column (300 mm × 7.8 mm, Bio-Rad) were equilibrated with 2.5 mM H_2_SO_4_ in water at 60°C and samples were eluted with 2.5 mM H_2_SO_4_ in water at a 0.5 ml/min flow rate. Data were acquired with Waters Millennium software.

Undissociated lactic acid was determined from supernatant samples diluted in ethyl acetate. The standard was prepared by dissolving lithium lactate in 0.5 M HCl and further diluting it in ethyl acetate. Samples and standards were eluted with the Fast Juice Column (50 mm × 7.8 mm, Phenomenex) and Fast Acid Analysis Column (100 mm × 7.8 mm, Bio-Rad) as above at 1.0 ml/min flow.

Lactate and ethanol yields were calculated as the amounts of accumulated products per amount of consumed sugar. Yields are reported for the sample time when sugar concentration was first observed to be below 1.5 g/l, unless otherwise stated.

An OD_600_ of 1 corresponded to 0.3 g/l cell dry weight.

Intracellular concentrations of lactic acid and pyruvate were measured from cells harvested from 1 ml of culture by centrifugation, washed with 1 ml 1 M Tris–HCl pH 9.0, resuspended in 1 ml of ice cold 5% (w/v) trichloroacetic acid by vortexing for 1 min and incubated on ice for 30 min. Samples were vortexed for 1 min, centrifuged at 13 000 rpm for 30 min at +4°C, and l-lactic acid in the supernatant was measured with the l-lactic acid UV method (#10139084035, Roche, Mannheim, Germany) method or by HPLC. Pyruvate was measured enzymatically using a pyruvate kit (Sigma Diagnostics, St. Louis, MO). Intracellular concentrations of lactic acid and pyruvate were calculated assuming that one gram of cell dry weight corresponds to 2 ml cell volume [34].

d-lactate was determined enzymatically with the l-lactate UV-method (#10139084035, Roche, Mannheim, Germany) using d-LDH instead of l-LDH in the assay.

### Statistical analyses

Data are given as means. Where appropriate, values were compared by analysis of variance (ANOVA) and significant differences determined using Fisher’s multiple range test. *P* values < 0.05 were considered statistically significant.

## Abbreviations

PDC: Pyruvate decarboxylase enzyme; PDC: Gene encoding for pyruvate decarboxylase; LhLDH: l-lactate dehydrogenase gene of *Lactobacillus helveticus*; BmLDH: l-lactate dehydrogenase gene of *Bacillus megaterium*; RoLDH: l-lactate dehydrogenase gene of *Rhizopus oryzae*; YP: 1% (w/v) yeast extract – 2% (w/v) peptone medium; X-α-Gal: 5-bromo-4-chloro-3-indolyl-α-D-galactopyranoside; TDH1: Gene for glyceraldehyde-3-phosphate dehydrogenase; PGK1: Gene for 3-phosphoglycerate kinase.

## Competing interests

PS is an employee of Cargill, which has financial interest in lactic acid producing microorganisms described here.

## Authors’ contributions

MI designed and carried out the molecular studies, participated in the cultivations, analysed the results, and drafted the manuscript. KK carried out the metabolite and enzyme analytics, participated in the cultivations and the analysis of results. LR and MP helped to draft the manuscript. VR and MP conceived of the study. MP, LR, PS and VR participated in its design and coordination. All authors read and approved the final manuscript.
